# Effects of the first lockdown of the COVID-19 pandemic on the trauma surgery clinic of a German Level I Trauma Center

**DOI:** 10.1007/s00068-021-01635-x

**Published:** 2021-04-15

**Authors:** Dirk Wähnert, Christian Colcuc, Georg Beyer, Markus Kache, Adrian Komadinic, Thomas Vordemvenne

**Affiliations:** 1 Protestant Hospital of Bethel Foundation, Department of Trauma Surgery and Orthopaedics, University Hospital OWL of Bielefeld University, Campus Bielefeld-Bethel, Burgsteig 13, 33617 Bielefeld, Germany; 2 Protestant Hospital of Bethel Foundation, Central revenue management, University Hospital OWL of Bielefeld University, Campus Bielefeld-Bethel, Kantensiek 11, 33617 Bielefeld, Germany

**Keywords:** COVID-19, Pandemics, Level 1, Trauma center

## Abstract

**Purpose:**

The effects of the first pandemic wave on a German Level I Trauma Center should be evaluated to find ways to redistribute structural, personnel, and financial resources in a targeted manner in preparation for the assumed second pandemic wave.

**Methods:**

We examined the repercussions of the first wave of the pandemic on the trauma surgery clinic of a Level I Trauma Center and compared the data with data from 58 other trauma clinics. The results could aid in orientating the distribution of structural, financial, and human resources (HR) during the second wave. The period between March 16 and April 30, 2020 was compared with the data over the same period during 2019. Information was collected from the HR department, central revenue management, and internal documentation.

**Results:**

The proportion of trauma surgical patients in the emergency room decreased by 22%. The number of polytrauma cases increased by 53%. Hospital days of trauma surgery patients in the intensive and intermediate care wards increased by 90%. The number of operations decreased by 15%, although the operating time outside of normal working hours increased by 44%. Clinics with more than 600 beds recorded a decrease in cases and emergencies by 8 and 9%, respectively, while the Trauma Center showed an increase of 19 and 12%. The results reflect the importance of level I trauma centers in the lockdown phase.

**Conclusion:**

To reduce the risk of an increased burden on the healthcare infrastructure, it suggests the care of trauma and COVID-19 patients should be separated locally, when possible.

## Background

With more than 78 million infected persons and over 1.7 million deaths worldwide the infected population with COVID-19 has surpassed the number reported during the SARS outbreak in 2003 and represents a major health crisis for the world population [1].

The virus was first detected in Germany at the end of January 2020. The measures to contain the spread of the virus have had direct and indirect impacts on all hospitals [2]. The Ministry of Health announced on March 12 2020, that elective and non-urgent operations in hospitals throughout Germany were to be postponed indefinitely. The aim was to accommodate strains on personnel and intensive care capacities for the treatment of seriously ill COVID-19 patients [3].

During this time, the trauma surgery clinics and in particular, the Level I Trauma Centers, with their care mandate for severely injured patients were facing a dilemma, between having to reduce surgical and intensive care capacities to ensure adequate emergency care [4–6]. Considering the dynamics of the pandemic and the current second wave of the disease, all available data and resources need to be used to prevent overloading and collapse of the health care system.

The already acquired and growing knowledge about COVID-19 should be used to achieve optimized prevention measures and efficient control of the supply routes. For this reason, the effects of the first pandemic wave should be evaluated to find ways to redistribute structural, personnel, and financial resources in a targeted manner during the second wave. The aim of this study was to examine the effects of the first wave of the pandemic on the trauma surgery clinic of a German Level I Trauma Center and to compare them with data from 58 other clinics. The Level I Trauma Center of a hospital with a total of 1750 beds, a catchment area of approximately 2.06 million inhabitants. On average, 140 polytrauma patients with a mean injury severity score of 26, are treated here annually.

## Materials and methods

For the analysis, the data of a German Level I Trauma Center for the period of March 16–April 30, 2020 (lockdown) were compared with data during the same period in 2019. Data from the human resources department, central revenue management and internal documentation were included in the analysis. To estimate the burden on hospital personnel, the total overtime of the clinic's nursing staff as well as doctors on the cut-off dates (29.02.2020 vs. 30.04.2020) were compared. Furthermore, patient contact for general and trauma visits in the central emergency room were evaluated as well as the number of polytrauma patients. The number of bed days of trauma surgery patients was evaluated for the station, intensive care unit (ICU), and intermediate care (IMC). The number of operations performed (outpatient and inpatient) with reference to urgency (based on the Timing of Acute Care Surgery classification TACS [7]) and time taken (within or outside the regular working hours) was also recorded. In addition, the performance figures (e.g. Case Mix [CM], Case Mix Index [CMI], and Day Mix Index [DMI]) of the clinic were also evaluated.

### Benchmark (Organized Management Information, InMed GmbH, Hamburg, Germany)

For comparison, InMed GmbH evaluated its database and made the anonymized results available to us. Therefore, in 2020, 166 German hospitals sent their data to the InMed GmbH. Taking into account the criteria listed below, data from 58 clinics can be included in the study. All cases of admission in the period from March 16 to April 30, 2019 and 2020 and discharge by May 31, 2019 and 2020 that had the characteristic of “trauma surgery” at discharge were examined. Furthermore, Chapter 19 (Injury, poisoning and certain other consequences of external causes) of the International Classification of Diseases (ICD)-10 Clinical Modification as well as Major Diagnostic Category (MDC) 08, 21A, 21B, 22 and PRE were used to filter the diagnoses. Hospitals have been distinguished by their size (< 600 and ≥ 600 beds). This is the maximum size that can be set to ensure anonymity. The mean number of beds in the group of hospitals < 600 beds was 485 beds. For the group ≥ 600 beds, it was 1.031 beds. The data evaluation was purely descriptive.

## Results

The results are summarized in Table 1.

**Table 1:** Summary of the results for the periods March 16th until April 30th in 2019 and 2020 with details of the percentage change between 2020 and 2019

Bed days trauma surgery patients	Observation period		%-change
	2019	2020	
ICU + IMC	201	382	+ 90
General ward	1913	1485	− 22
**Emergency department**			
Cases overall	2481	1889	− 24
Cases trauma surgery	690	536	− 22
Cases trauma outpatient	477	336	− 30
Cases trauma inpatient	173	153	− 12
Cases polytrauma (ISS ≥ 16)	15	23	+ 53
Mean ISS polytrauma	32	30	− 6
**Surgeries**			
Number trauma surgeries	276	235	− 15
Surgical time (h)	443.485	388.764	− 12
Number surgeries inpatient	242	219	− 10
Number surgeries outpatient	34	16	− 53
Surgical time—regular working hours	73.2%	61.3%	− 16
Surgical time—outside regular working hours	17.3%	24.8%	+ 43
Surgical time—weekends + public holidays	9.5%	13.9%	+ 46
Surgery—elective	77.2%	50.2%	− 35
Surgery—urgent (within 24–48 h)	5.1%	22.1%	+ 333
N3 (surgery same day)	11.2%	16.6%	+ 48
N2 (surgery in next available trauma theater)	4.7%	5.5%	+ 17
N1 (surgery in any next available theater)	1.4%	5.1%	+ 264
N0 (surgery immediately)	0.4%	0.4%	0
**Department for trauma surgery and orthopedics**			
Number of cases	286	236	− 17
Case mix	403.746	316.668	− 22
Case mix index	1.412	1.342	− 5
Day mix index	0.207	0.223	+ 7
Patient clinical complexity level (PCCL)	0.8	0.9	+ 8
Age	57.6	58.0	–
Length of stay	6.8	6.0	− 12

### Personnel

The workload of the staff decreased, as measured by overtime during the lockdown. The nursing staff of the general wards were able to reduce their overtime by 34%, and the doctors, by 17%.

### Bed days

The cumulative number of ICU and IMC beds occupied by trauma surgery patients increased by 90% during the lockdown period compared to the same period last year. In contrast, the number of bed days of trauma patients in the general ward fell by 22%. None of the trauma surgery patients during the study period were positive on admission or have been tested positive during hospital stay for COVID-19.

### Clinic—emergency department

In the central emergency department, the total number of patient contacts fell by 24% and the number of trauma surgery contacts, by 22%. Inpatient admissions from the emergency department decreased by only 9%. In contrast, the number of polytrauma cases increased by 53% (mean ISS 2019: 32, 2020: 30).

### Clinic—operating theater

The number of operations performed fell by 15% in 2020, whereas outpatient operations were strongly affected (− 53%). The time spent in surgery (beginning to end of surgical procedures) during regular working hours decreased by 16% in 2020 but increased by 44% outside regular working hours.

### Clinic—total

The total number of cases at the clinic decreased by 17%, due to a 50% reduction in elective admissions by the hospital. The number of emergency cases fell by only 2%. The CMI fell by 5%, whereas the DMI and Patient Clinical Complexity Level (PCCL) increased by 7 and 8%, respectively.

### Benchmark

The results of the benchmark analysis are summarized in Table 2. For the clinics with < 600 beds, the lockdown had no significant impact on case numbers, but the length of stay and PCCL decreased by 9 and 7%, respectively. The clinics with ≥ 600 beds recorded an average decrease in cases and emergencies of 8% and 9%, respectively, due to the lockdown. The PCCL fell by 5%, while the length of stay increased by 13%. The evaluated Trauma Center recorded an increase in cases and emergencies of 19 and 12%, respectively; the PCCL increased by 20% and the length of stay, by 38%.

**Table 2:** Benchmark analysis of 58 trauma clinics compared with the evaluated Level I trauma center

	Hospitals < 600 beds (MW per clinic)			Hospitals ≥ 600 beds (MW per clinic)			Level I trauma center (1750 Bettenbeds)		
	2019	2020	%-change	2019	2020	%-change	2019	2020	%-change
Cases	68	69	1	105	96	− 8	95	113	19
Emergency cases	67	68	1	102	93	− 9	91	102	12
CM	107.89	91.48	Not comparable due to change in DRG algorithm	207.64	175.87	Not comparable due to change in DRG algorithm	181.46	289.06	Not comparable due to change in DRG algorithm
CMI	1.58	1.33		1.99	1.83		1.91	2.56	
DMI	0.20	0.19		0.20	0.16		0.24	0.23	
Length of stay	7.84	7.12	− 9	9.97	11.27	13	7.91	10.95	38
Age	64.44	65.65	2	64.24	65.71	2	56.93	57.88	2
PCCL	0.82	0.76	− 7	1.11	1.06	− 5	1.11	1.33	20

Grouping according to bed size, presentation as mean value (MW) per clinic

### Compensation payment

The overall financial impact and impact on the compensation payment were considered according to the method of Dercks et al. [8]. There was an underfunding of €1954.51 per case for the evaluated Trauma Center and thus a deficit of €97,725.49 for the period of the lockdown. After adjustment for non-accruing material costs, the shortfall was €526.09 per case and €26,304.63 for the entire lockdown period.

## Discussion

The global COVID-19 pandemic is having a profound impact on all areas of life. Social restrictions are necessary to contain the transmission of the virus. Hospitals, in terms of both outpatient and inpatient care, must face the new challenges in the short term and develop interdisciplinary solutions to find a balance between emergency care and the economically important elective care. Given the unpredictable dynamics of the event and the risk of a second pandemic wave, it is important to evaluate hospital data recorded in the first wave to be able to take appropriate measures in the future.

The early German governmental interventions in the form of a lockdown in the first pandemic wave made it possible to control the overload on health care system. Considering that accidents and serious injuries are not subject to pandemic-related restrictions on health care [5, 6], a second wave with increasing case numbers and a confusing infection situation in the population would most likely lead to an intensification of the effects described here. Accidental injuries are important social and health issues and major causes of death and disability [6]. While Trauma Centers care for patients with polytrauma and serious injuries, they also have to undertake additional tasks of accident prevention and education, as well as innovation and research [6].

We found that personnel burdens on the trauma surgery sector were reduced during the first lockdown due to the fall in the numbers of elective consultation hours and elective surgery patients. This fact is contrasted by a 50% increase in the number of polytrauma patients. There was a 90% increase in the number of bed days of trauma surgery patients in the ICU and IMC during the first lockdown as compared to the same period last year. Thus, in addition to the care of COVID-19 patients, there was also the additional burden of trauma patients on intensive care and monitoring capacities.

In addition, the combination of the increased number of trauma patients and the reduction in surgical contingency resources in the daily operations of the hospitals led to an increase in surgical activities outside of regular working hours. Additionally, the increase in urgent surgery cases is mainly due to the fact that every fracture with the indication of surgical treatment becomes an urgent operation after a few days. Working hours had increased by 46% on weekends and public holidays and by 43% on regular days.

These data are specific to the situation of Germany and are not transferable. However, they do show a trend that should be taken into account in personnel planning in the future.

In their international study, Greenhalg et al. showed that in a British national Trauma Center, the number of trauma surgery patients fell by 51% during the lockdown. Furthermore, the number of trauma surgeries performed fell by 43% and the number of polytrauma cases fell by 35% compared to in the same period last year [9]. This trend was also confirmed by Park et al., who investigated the effects of the COVID-19 pandemic on a Level I Trauma Center in London; there was a reduction of approximately 46 and 37% in trauma surgery cases and surgical interventions. However, the number of polytrauma cases was found to be constant with 18 cases in 2019 and 17 cases in 2020 [10].

A study by Lubbe et al. on the effects of “social distancing” on a Level I Trauma Center in the southwest of the USA showed that the number of patients in the lockdown period fell significantly compared to the corresponding periods in 2018 and 2019. The number of polytrauma cases did not show any significant difference. However, in 2020, the authors found a significant increase in the waiting time until hospital visit and a significant decrease in the length of stay [11]. The results from a regional trauma center in the USA also showed a significant decrease in trauma surgery patient contact by 45% [12].

Wong et al. analyzed the trauma surgery/orthopedic treatments in 43 public hospitals and 122 outpatient clinics in Hong Kong and compared data from January 25 to March 27, 2020 (COVID-19 cohort) with data from the same period from 2016 to 2019; they found a 73.5% decline in elective surgery and 21.2% decline in emergency surgery numbers. One study on accident and emergency contacts showed a significant reduction in 2020 in all areas: sports accidents (− 59.3%), accidents at work (− 45.6%), household accidents (− 38.2%), and traffic accidents (− 26.4%). However, there no further differentiation according to injury severity was made [13].

Accidents and injuries, especially polytrauma, can only be influenced to a small extent. Reductions from 0 to 35% have been reported. Locking at the results of 58 German trauma clinics the decrease in cases (8%) and emergency cases (9%) more or less reflects the available literature. In contrast, the length of stay increased in the hospitals ≥ 600 beds by 13%, whereas the PCCL decreased by 5%. Lubbe et al. described a significant decrease in the length of stay comparing the lockdown 2020 with the same period in 2018 [11]. There was a significant increase in the number of polytrauma cases in the German Trauma Center investigated, which may be due to local peculiarities (e.g. the only supraregional trauma center in the region, COVID staff shortages in surrounding clinics). In the observation period of 2020, the number of polytrauma cases was above average, whereas the number in 2019 was average.

Another important aspect to consider is how the COVID-19 pandemic will affect trauma centers financially. Maintaining the structure of the trauma centers is a personnel and cost-intensive task. If a decrease in the number of polytrauma patients as shown in the above-mentioned studies, should become apparent in the long term throughout Germany and if compensation through elective interventions is not possible due to the pandemic, trauma centers will face a considerable financial shortfall [6].

The results of this study reflect the special position of Level I Trauma Centers during the lockdown and pandemic. There is a risk of increased burden on the infrastructure, especially in the central emergency departments, intensive care, anesthesia, and surgical units. Finding the balance between treating patients with confirmed or suspect COVID-19 infection and ensuring the provision of sufficient trauma care is difficult, and thus pushing the personnel resources of a clinic to their limits. The data presented here should be taken into account in the planning of future pandemic periods.

One solution would be to separate trauma patients and COVID-19 patients in larger cities with appropriate hospital infrastructure. In this way, personnel and infrastructure resources could be bundled and used effectively.

Further studies can examine whether the tiered structure of trauma networks (local, regional, supraregional) has led to the increased use of higher levels of care during the pandemic [14].

This study is limited by the short observation period and the local specificity of the network structures so the data cannot be generalized to other networks. Furthermore, the data evaluation was purely descriptive and the comparison with the 58 clinics only allows a rough classification, as more precise limitations are not applicable due to the anonymous data analysis.

## Conclusion

To reduce the risk of an increased burden on the healthcare infrastructure, it suggests the care of trauma and COVID-19 patients should be separated locally, when possible.

## Data Availability

The datasets used and/or analysed during the current study are available from the corresponding author on reasonable request.
